# Immunology in COPD and the use of combustible cigarettes and heated tobacco products

**DOI:** 10.1186/s40001-023-01374-2

**Published:** 2023-10-04

**Authors:** Justyna Błach, Mateusz Siedliński, Wojciech Sydor

**Affiliations:** 1 Department of Clinical Immunology, UCH, Cracow, Poland; 2 Department of Internal Medicine and Rural Medicine, Jagiellonian University Medical College, Cracow, Poland; 3 Department of Rheumatology and Immunology, Jagiellonian University Medical College, Cracow, Poland

**Keywords:** Chronic obstructive pulmonary disease, Heated tobacco products, Combustible cigarettes, Lung immunology

## Abstract

Chronic obstructive pulmonary disease (COPD) is one of the most common chronic respiratory diseases, characterised by high morbidity and mortality. COPD is characterised by a progressive decline of lung function caused by chronic inflammatory reactions in the lung tissue due to continual exposure to harmful molecules by inhalation. As prevention plays a very important role in COPD, quitting smoking is the most important factor in reducing the decline in lung function. Unfortunately, many people are unable to break their nicotine addiction. This paper summarises current knowledge about combustible cigarettes (CSs) and alternative tobacco products such as heated tobacco products (HTPs) in COPD. The paper focuses on the immunological aspects of COPD and the influence of tobacco products on lung tissue immunology. There are differences in research results between HTPs and CSs in favour of HTPs. More long-term studies are needed to look at the effects of HTPs, especially in COPD. However, there is no doubt that it would be best for patients to give up their nicotine addiction completely.

## Introduction

Chronic obstructive pulmonary disease (COPD) is a progressive lung disease caused by the inhalation of harmful chemicals. These chemicals cause inflammation in the lung tissue, resulting in lung damage and increased mucus production in the airways [1]. It is estimated that COPD worldwide prevalence is 9–10% in adults over 40 years of age. In 2017 alone, up to 3.2 million people died from COPD, and it is estimated that by 2040 this number will reach 4.4 million per year. Smoking is believed to be the main risk factor for COPD, although genetic factors, and especially α1-antitrypsin deficiency, play a significant role in COPD development as well [2]. Looking at the statistics from a smoking perspective, one in five smokers will develop COPD and almost half of COPD deaths are regarded as being caused by smoking [3, 4]. There are still problems with the diagnosis of COPD, which is mainly considered a disease of the elderly only [5]. The diagnosis of COPD is confirmed by spirometry when the post-bronchodilator ratio of forced expiratory volume in 1 s (FEV1) to forced vital capacity (FVC) is < 0.7 [6]. Disease progression, as assessed by a decline in FEV1 value, is closely related to active smoking [7]. Early-age diagnoses allow for earlier interventions, such as quitting smoking, which may normalise the worsening of lung function [5].

COPD is considered a chronic disease; however, exacerbations, which occur in a significant number of patients, are an important problem. A COPD exacerbation is clinically defined as an increase in breathlessness, cough, or purulent sputum with or without evidence of an upper respiratory tract infection [8]. Acute exacerbations of COPD significantly affect the deterioration of lung function and are the cause of increased mortality [2, 9]. Factors that contribute to the occurrence of exacerbations include smoking, environmental pollution, airway infections and multiple comorbidities [9–11]. It is important to prevent or at least reduce the incidence of COPD exacerbations.

There are two common, partially overlapping processes in COPD. The first is the disease of the small airways, which affects obstructive bronchiolitis, remodelling of the airways and narrowing of the peripheral airways. The second is emphysema, with the destruction of the respiratory bronchioles, air trapping and hyperinflation [12]. Knowledge of the pathophysiology of COPD is constantly expanding. It appears that the phenotype and disease progression may differ in COPD caused by exposure to biomass fuel compared to COPD caused by tobacco smoking. COPD due to exposure to biomass is mainly related to the thickening of the airway walls and improvement in lung function following bronchodilator therapy. Smoking-induced COPD is characterised by more severe emphysema and a faster decline in lung function [13].

It is clear that cigarette smoking contributes to the development of many diseases, including COPD. New products have been developed to deliver nicotine in a way that appears to be less harmful. Currently, there are alternative products to conventional cigarettes (combustible; CSs), such as electronic cigarettes (e-cigarettes) or heated tobacco products (HTPs). Electronic cigarettes refer to personal vaporisers that generate an aerosol by heating a liquid containing nicotine. HTPs operate by heating the tobacco through a battery-supported device that heats the tobacco in a controlled manner. The temperature generated by HTPs is 330–350 °C, which is much lower compared to the combustion temperature of 850 °C at the tip of the cigarette. The assumption is that the aerosol delivered to the lungs in this process should contain significantly lower concentrations of toxic substances than cigarette smoke [14, 15]. There are discussions about whether non-combustible tobacco products can be a tool to help people quit smoking and whether they are a less detrimental choice than cigarettes.

In this article, we will focus on the immune response in COPD to give an in-depth understanding of the mechanisms that lead to lung tissue damage. Then, based on them, as the data are available, we will compare the effects of smoking on lung tissue with the accessible information on alternative products, especially HTPs.

## Immunology in COPD

In COPD, chronic inflammation of lung tissue is observed and is associated with abnormal immune responses leading to increased tissue damage and subsequent lung remodelling [16]. Inflammation in COPD is mainly caused by a type 1 and type 3 immune response. Type 1 is antimicrobial and involves type 1 helper cells (Th1), T-cytotoxic cells (Tc) and group 1 innate lymphoid cells, and the transcription factor T-bet, which regulates the secretion of IFNγ. The activation of pro-inflammatory macrophages is also increased. The type 3 response is mainly directed against fungi and is coordinated by type 17 helper T cells (Th17) and group 3 innate lymphoid cells that express RORγt and secrete IL-17 and IL-22, leading to neutrophil inflammation [17]. These mechanisms are described in more detail below, divided into specific and non-specific responses.

### Innate immunity

The innate response is the body’s first defence mechanism, consisting of physical barriers and specialised cells of the immune system. The first element activating the innate response is the recognition of antigens belonging exclusively to microorganisms—pathogen-associated molecular patterns (PAMPs). These antigens are recognised by the so-called pattern recognition receptors (PRRs), as expressed in dendritic cells, macrophages, monocytes, and neutrophils, as well as in epithelial cells, endothelial cells and fibroblasts [18]. There are several classes of PRR, such as Toll-like receptors (TLRs), cytosolic NOD-like receptors (NLRs), RIG-I-like receptors, and C-type lectin receptors [19]. Activation of the non-specific inflammatory response is also triggered by damage to epithelial tissue and the release of endogenous molecules, called danger-associated molecular patterns (DAMPs). Inhalation of toxic and irritating agents, infections, oxidative stress and tissue hypoxia, leads to the release of DAMPs through the damaged respiratory tract. These molecules are recognised by PRRs such as Toll-like receptors 2 and 4 on epithelial cells, thereby releasing pro-inflammatory cytokines [20]. Contact with cigarette smoke leads to the activation of PRRs, directly by individual components of cigarette smoke and by damaging the epithelium from which DAMPs are released [21].

Cytokines are molecules that activate and control immune response. Cytokines are produced by innate and adaptive immune cells, as well as structural cells (epithelial and mesenchymal cells). They can be divided into pro-inflammatory (TNF-α, IFN-λ, IL-1, IL-6, IL-8, IL-13) and anti-inflammatory cytokines (IL-10, TGF-β). Cigarette smoke influences the release of pro-inflammatory cytokines and chemokines, such as tumour necrosis factor α (TNFα), by the airway epithelium and alveolar macrophages, resulting in an influx of neutrophils and monocytes into the lung tissue [22]. The number of neutrophils and macrophages is increased in the lungs of smokers and COPD patients. Activated neutrophils and macrophages release oxygen radicals and proteolytic enzymes such as neutrophil elastase and matrix metalloproteinases (MMPs), including MMP-8, MMP-9, and MMP-12 [23]. Proteolytic enzymes and reactive oxygen species contribute to tissue damage [20]. The function of neutrophilic elastase is also to induce mucin production and secretion. This process occurs by cleavage of transforming growth factor α (TGFα), which is a ligand for the epidermal growth factor receptor. Excessive mucus production and the resulting obstruction of the airway are observed in COPD patients [24].

The role of alveolar macrophages is the phagocytosis of neutrophils involved in the inflammatory response. This process aims to regulate the immune response and inhibit excessive inflammation [25]. However, this regulatory mechanism is disturbed in COPD. Despite the large number of macrophages infiltrating the lung tissue in COPD, their phagocytic capacity in smokers is reduced, and the neutrophil load of the airways is increased [26]. CSs also influence the differentiation of alveolar monocyte precursors to the M2 macrophage phenotype. Research shows that increased numbers of M2 macrophages secreting metalloproteinases contribute to FEV decline, disease progression and severity [27].

**Fig. 1:**
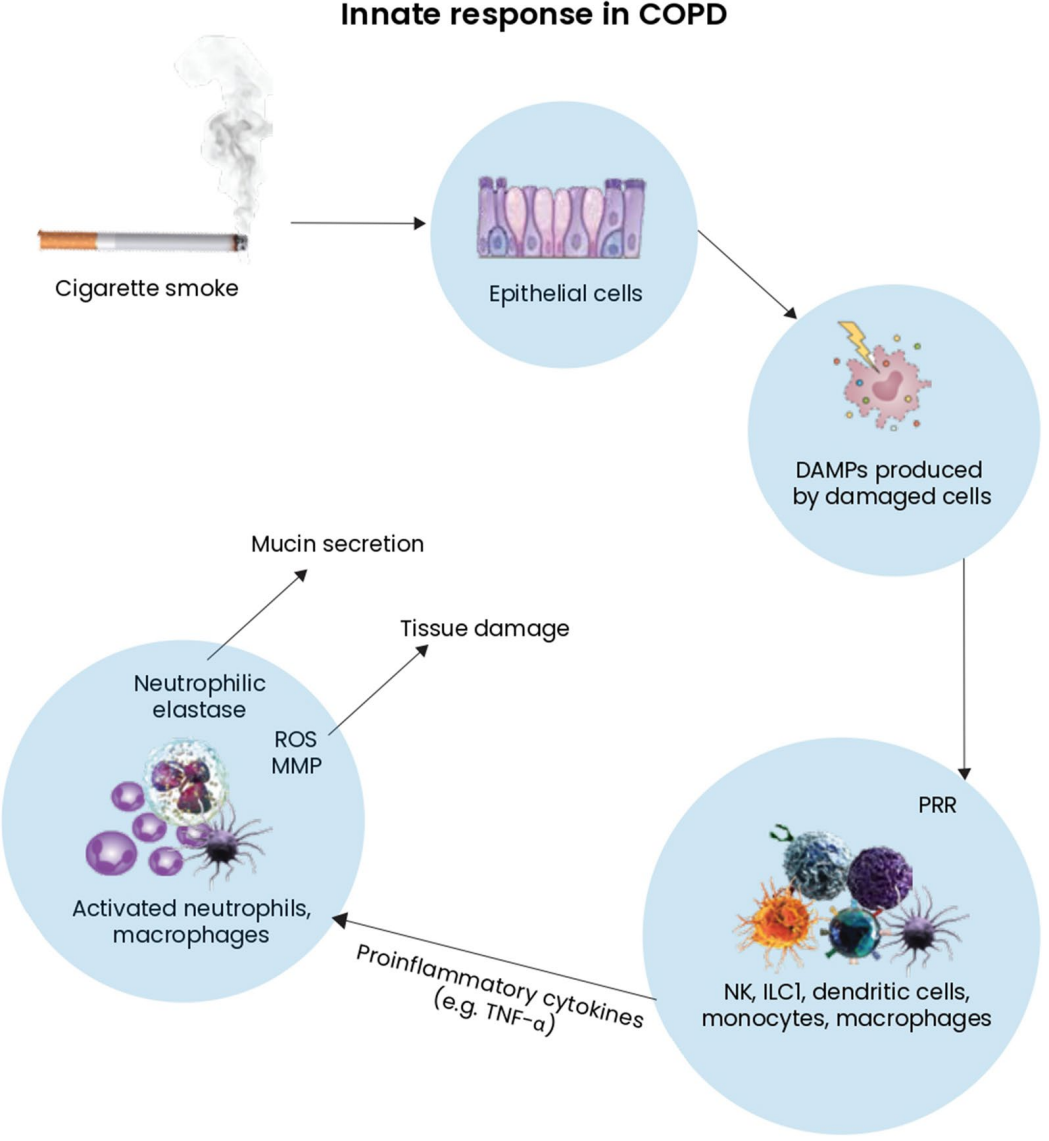
The mechanisms of the innate immune response in COPD. *DAMPs* danger-associated molecular patterns, *ROS* reactive oxygen species, *MMP* matrix metalloproteinases, *TNF* tumour necrosis factor, *NK* natural killer, *ILC* innate lymphoid cells

Langerhans-like dendritic cells expand on the surface of the airway epithelium in smokers. Upon exposure to tobacco smoke, myeloid dendritic cells are immediately detected in the bronchoalveolar lavage fluid [28]. The mechanisms of the innate immune response are shown in Fig. 1.

### Adaptive immunity

Innate immune cells activate an adaptive immune response. The main populations of specific response cells are T cells (including CD4+ and CD8+), responsible for the cellular response and antibody-producing B cells. Histological studies of lung tissue show that remodelling and destruction of bronchiolar and alveolar tissue are associated with excessive infiltration of macrophages, CD4, CD8 and B cells, and the formation of tertiary lymphoid organs. Activated CD8 T cells release perforins and granzymes, and these proteolytic enzymes cause infected or tumour cell death through apoptosis or necrosis [29].

Inflammation is exacerbated by Th1 cells producing interferon gamma and Th17 secreting IL-17 in response to damage of the epithelium exposed to cigarette smoke [30]. Studies have shown that COPD patients’ lung tissue is also rich in innate lymphoid cells 1, NK cells and lymphoid tissue-inducer cells. These cells induce a Th1-dependent response and contribute to emphysematous destruction in COPD [31].

Moreover, it has been found that smokers also have significantly fewer lung T-regulatory cells responsible for extinguishing inflammation and protecting against autoimmunity. The development of autoimmunity has been reported in patients with severe COPD [32].

**Fig. 2:**
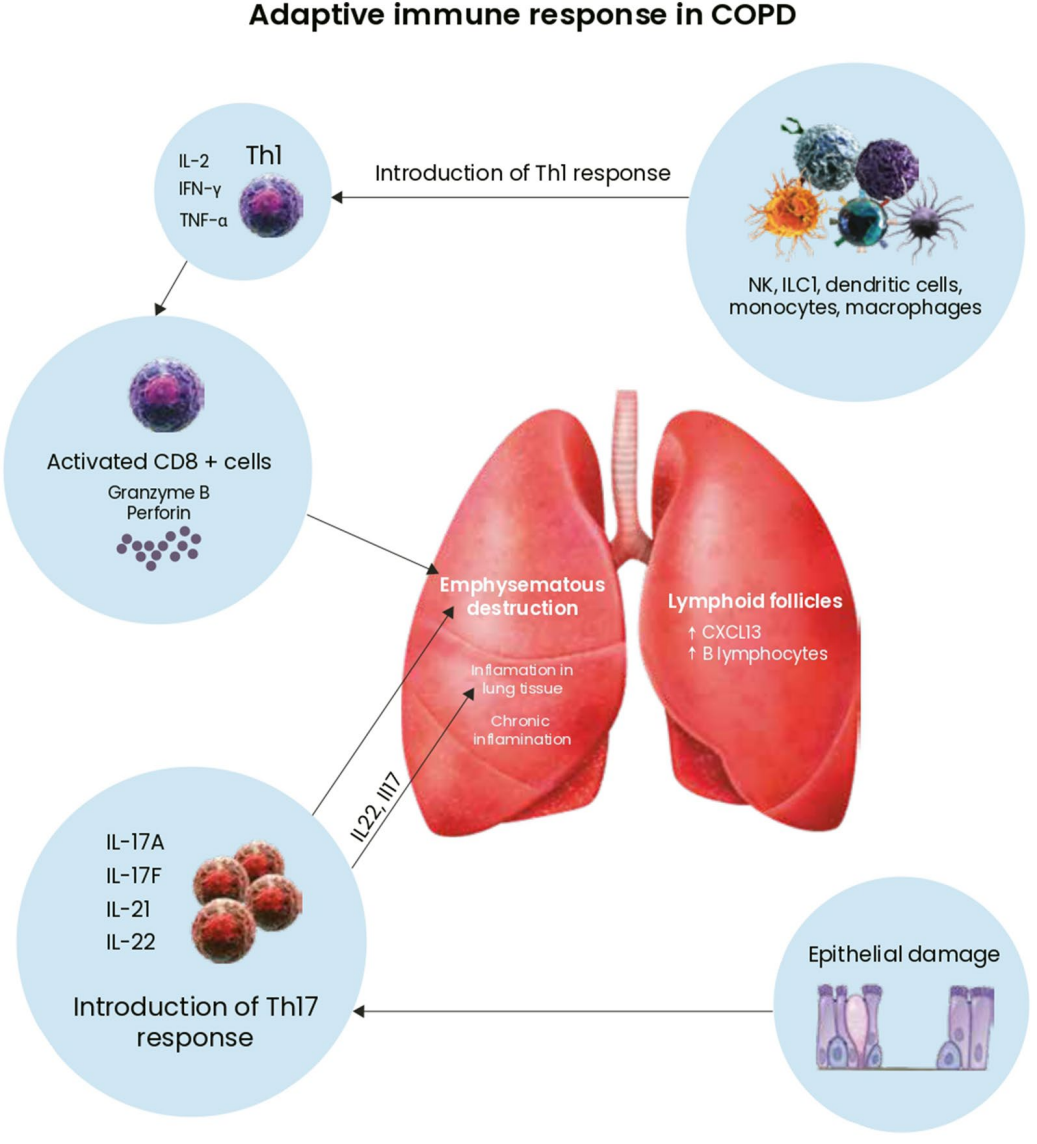
The mechanisms of the adaptive immune response in COPD. *IL* interleukin, *IFN* interferon, *TNF* tumour necrosis factor, *Th* T helper cells, *NK* natural killer, *ILC* innate lymphoid cells

B lymphocytes in the large airways are increased in COPD patients [33]. Lymphoid follicles are observed around the smaller airways as a result of lymphoid neogenesis due to chronic inflammation [34]. The formation of the above-mentioned follicle may be related to a chemokine CXCL-13-dependent mechanism, which engages Toll-like receptor and lymphotoxin receptor signalling. Cigarette smoke, H2O2, and exposure to lipopolysaccharide raise the levels of B cell-derived CXCL13 [35]. Chronic bronchitis has been observed for years after smoking cessation in COPD patients. Ongoing adaptive immune response contributes to this phenomenon [20]. The mechanisms of the adaptive immune response are shown in Fig. 2.

## Genetic links between nicotine addiction, immunological response and COPD

While smoking is a major risk factor for COPD development, both lung function and nicotine addiction are at least partially complex heritable traits. Smoking induces epigenetic changes in COPD patients, and genome-wide association studies (GWAS) have provided compelling evidence on the association between polymorphisms in *CYP2A6*, a nicotine metabolising enzyme, on chromosome 19 and nicotinic acetylcholine receptors (*CHRNA3/CHRNA5/CHRNAB4*) cluster on chromosome 15 with the number of cigarettes smoked per day both in the general population and COPD patients [36, 37]. Moreover, GWAS in over 400,000 subjects found over 270 independent loci significantly associated with lung function parameters, such as FEV_1_, FVC, PEF and FEV_1_/FVC ratio, an indicator of airflow obstruction [38]. Some of these loci may support the existence of a direct link between immunology and COPD, and these include Advanced Glycosylation End-Product Specific Receptor (AGER), human leukocyte antigen class II DQ, beta chain locus 1 (HLA-DQB1), IL27 [39]. AGER is considered a key pathway in the pathophysiology of COPD. The effect of smoking on AGER gene expression is investigated [40]. Following the pattern of the distribution of the HLA class II allele in autoimmune diseases, the distribution of the HLA-DQB1 allele in COPD patients was investigated. No significant dependency was found [41]. In the coding variation in the COPD study, an IL27 variant, rs181206, was identified to be associated with PCOHP susceptibility. It is at a locus that has previously been correlated with genetic variants associated with diabetes, inflammatory bowel disease, and obesity [42].

## Cigarettes versus HTPs

Based on pathophysiological knowledge, studies have been carried out to determine how the inflammatory response in the lungs changes due to the use of different types of tobacco products. A pilot study was conducted by Leigh et al. in which bronchial epithelial cells were directly exposed in vitro to HTP, e-cigarette and tobacco emissions compared to the control air. In cytotoxicity tests assessing the viability and metabolic activity of cells, HTPs were found to exhibit higher cytotoxicity than the control air. HTPs also exhibit higher cytotoxicity than e-cigarettes, but lower than combustible cigarettes. The levels of secreted cytokines commonly used as inflammatory markers were also tested using an ELISA test. It was observed that bronchial epithelial cells exposed to HTP emission released less IL-1β and IL-6 than cells exposed to cigarette smoke. Moreover, no differences in cytokine levels were found between the HTPs versus e-cigarettes [43]. Another study compared the effects of CSs, HTPs and e-cigarette exposure on the lungs of type II diabetic mice compared to non-diabetic mice. The animals were exposed for 6 h/day for 7 days, and the control group was exposed to air. Lung damage was assessed based on various markers, including expression of TNF-α, IL-6, and IL-1 β, reactive oxygen species production (ROS), and assessment of cell apoptosis. The expression of inflammatory mediators in lung tissue was generally higher in the diabetic groups. When comparing inflammatory mediators in the non-diabetic groups, it was found that HTPs did not increase inflammation significantly, unlike CSs. The level of TNF-α, IL-6, and IL-1 β expression was increased in both groups (with and without diabetes) during the use of CSs, while with HTPs, IL-1β and TNF-α increased significantly only in the diabetic group. In the e-cigarette non-diabetic group, the level of IL-1 β significantly increased. HTP exposure did not cause any significant oxidative stress in either the diabetic or non-diabetic groups. The production of ROS was significantly increased following the use of CSs (with greater intensity in the group with diabetes). Similar results were obtained when evaluating the number of apoptotic nuclei indicating cell death. Exposure to HTPs did not cause any significant increase in apoptosis [44]. This study was also important as it showed the effects of various tobacco products on the lungs in the presence of other diseases, such as diabetes, one of the most prevalent diseases in the population.

Subsequent studies that should be mentioned have quite different results. Bhat et al. investigated the effect of short-term exposure to HTPs and CSs in mice. They found that the total number of lung infiltrating leukocytes was equivalent after exposure to both aerosol from HTPs and CSs smoke, but it was significantly increased compared to air-exposed controls. Additionally, the number of CD4+RORγt+T cells was significantly increased in both groups. The RORγt receptor regulates the development of Th17 lymphocytes and influences the development of autoimmune diseases and inflammation. Researchers also found an increase in pro-inflammatory cytokines. Their conclusion was that HTPs and CSs cause similar damage and pro-inflammatory changes in the lungs [45]. In contrast, in a study by Wong et al., accumulation of macrophages, neutrophils, and CD4+ and CD8+ lymphocytes in the lungs, as well as significantly elevated levels of inflammatory mediators in BALF were noted in CS-exposed mice but not in HTP aerosol-exposed mice. Similarly, altered lung function and emphysematous changes were observed only in CS-exposed mice [46]. The results of independent studies conducted by Bhat are different from the results of Wong. However, it should be noted that in Bhat’s study, the exposure to HTP aerosol was unreasonably high, and this may explain the toxic effects.

ROS initiate oxidative stress and cause the secretion of inflammatory cytokines that stimulate the development of chronic inflammation, resulting in the remodelling of the airways. This mechanism is a trigger for many smoking-related diseases. Salman et al. showed that switching from classic cigarettes to HTPs reduces the intake of particulate ROS by 82% and gaseous ROS by 90% [47].

Mitochondrial dysfunction, caused by cigarette smoke, leads to oxidative stress. There is a concept that heating tobacco instead of smoking can reduce the levels of harmful ingredients and, therefore, can reduce mitochondrial dysfunction that is associated with cell damage [48].

Sohal et al. studied the effects of exposure to classic cigarettes, HTPs and e-cigarettes on cells in vitro. They showed the release of the CXCL8 chemokine from human bronchial epithelial cells and primary human airway smooth muscle cells following the cells’ treatment. It was also found that exposure to all tobacco products increased the release of collagen 1 and fibronectin in a concentration-dependent manner. Exposure to the above products also contributed to a change in mitochondrial function and an increase in extracellular acidification. Based on these results, it was concluded that products such as e-cigarettes and HTPs, similar to classic cigarettes, can increase oxidative stress, inflammation and airway reconstruction [49]. In this study, the toxicity threshold was not established, so it is not known whether the toxicity of HTPs compared to conventional cigarettes is due to the overexposure of cells.

There is some data available on the risk assessment of allergic diseases and the use of tobacco products. A study by Lee et al. involving a large group of students showed that the use of any tobacco product (CSs, e-cigarettes and HTPs) was significantly associated with an increased risk of asthma, allergic rhinitis and atopic dermatitis. However, it was a self-reported study without validation on clinical measurements (e.g. serum IgE levels or eosinophil counts) nor questions on the frequency and severity of symptoms [50]. Nonetheless, this study indicates that it is important to note that smoking any tobacco or nicotine-containing product may contribute to the development of allergic diseases. It should also be mentioned that cases of acute eosinophilic pneumonia (AEP) following the use of HTPs have been reported. Until now, cigarette smoking has been a known cause of AEP that developed within weeks of starting smoking. These cases may show that allergic reactions in the lungs are not suppressed after contact with the particulate produced by HTPs [51–53].

## Toxicant emission

The main toxicants affecting the respiratory system include ozone (O_3_), carbon monoxide (CO), particulate matter (PM2.5, PM10) and sulfur dioxide (SO_2_). They cause oxidative stress and inflammation that damage the airways [45]. In recent years, more attention has been paid to the influence of particulate matter (PM) on the incidence of respiratory diseases, especially the risk of exacerbation of COPD. PM is composed of solid and liquid particles, classified according to its aerodynamic diameter as PM10 (< 10 μm, coarse particles), PM2.5 (< 2.5 μm, fine particles), and PM0.1 (< 0.1 μm, ultrafine particulates). Larger PM10 particles settle in the upper respiratory tract, causing allergic and irritating reactions. The smaller ones, like PM2.5, enter the terminal bronchioles and alveoli and are small enough to enter the bloodstream across the blood–air barrier [54]. PM2.5 reduces the defences of the airway epithelium and changes the immune response. They affect mucociliary movements and increase mucus production and reduce the production of antimicrobial proteins such as beta-defensins [55, 56]. PM2.5 may also interfere with the phenotype and function of alveolar macrophages, and impair the function of other important immune cells such as neutrophils, NK cells and lymphocytes [56–59]. Alveolar macrophages produce pro-inflammatory mediators upon phagocytosis of PM, leading to oxidative stress and systemic inflammation [60]. PM increases the production of ROS, which may result in cell and tissue damage, mainly through DAMPs and PAMPs-mediated TLR4 activation [61].

The emissions of PM in confined spaces were assessed when smoking traditional cigarettes and using non-combustible tobacco products. Peruzzi et al. showed in the randomised trial that using non-combustible tobacco products leads to significantly lower levels of indoor PM in comparison to classic cigarettes [62]. A similar study was performed by Protano et al. where PM with an aerodynamic diameter smaller than 10, 4, 2.5 and 1 µm (PM10, PM4, PM2.5, PM1) were measured indoors after using alternatives to conventional cigarettes. The study showed that the aerosol from HTP consisted mainly of PM1 (> 95%). As in the above study, alternative products gave lower concentrations of PM1 than conventional cigarettes. However, they showed that all tested products deteriorate indoor air quality. This is essential information because the size of the measured aerosol was mainly below 1 μm, and therefore was able to penetrate deeply and effectively into the respiratory system [63].

Various studies, dependent and independent of the tobacco industry, have been carried out to compare the toxicant emissions of conventional cigarettes and HTPs. It is estimated that cigarettes contain about 5000 toxic substances such as nicotine, tar, carbon monoxide, volatile organic compounds (VOCs), polyaromatic hydrocarbons (PAHs) and tobacco-specific nitrosamines (TSNAs) [64]. Due to the lower heating temperature of the tobacco in HTP systems compared to combustion in conventional cigarettes, less exposure to toxic substances is suspected. Independent studies have shown that the concentration of harmful chemicals produced by HTPs is lower than in traditional cigarettes [65, 66]. Studies have also shown that HTPs contain lower levels of nicotine than conventional cigarettes [14, 65, 67].

Farsalinos et al. confirmed that HTPs emit significantly lower levels of carbonyls compared to commercial tobacco cigarettes with the 3 puff patterns tested, but more compared to e-cigarettes. In their study, the levels of major carbonyls for HTPs were, on average, 91.6% lower for formaldehyde, 84.9% lower for acetaldehyde, 90.6% lower for acrolein, 89.0% lower for propionaldehyde and 95.3% lower for crotonaldehyde, compared to cigarettes [66]. Dusautoir et al. compared the composition of emissions from HTPs, e-cigarettes and conventional cigarettes in terms of selected harmful or potentially harmful compounds and their toxic effects on human BEAS-2B bronchial epithelial cells. They confirmed previous reports that HTPs emit fewer carbonyls and polycyclic aromatic hydrocarbons than a cigarette, but more than an e-cigarette. They also found that increasing the potency of e-cigarettes affects the level of toxic compounds and the associated oxidative stress [68]. Mallock et al. also noticed that the HTP emission of aldehydes is lower by about 80–95%, and other volatile organic compounds even by about 97–99% compared to the emissions of these substances when smoking combustible cigarettes [65].

## Clinical outcomes

It is important to consider independent observational studies that have been conducted on patients with COPD. A 3-year study by Polosa et al. included patients with COPD who gave up traditional cigarettes or significantly limited their use in favour of HTPs. They were compared to patients of the same age and sex who continued smoking. Patients were assessed at 12, 24 and 36 months. Changes in daily smoking, the number of disease exacerbations, lung function indexes, patient-reported questionnaires (COPD Assessment Test—CAT), and 6-min walk distance (6MWD) from baseline were assessed. A significant reduction in the number of annual exacerbations was found in patients using HTPs (*p* < 0.05). Additionally, at all three time points in the HTP group, there was a clinically significant improvement in CAT and 6MWD scores. No significant changes were seen in COPD patients who continued to smoke. As mentioned in the introduction, exacerbations significantly contribute to disease progression and mortality; therefore, their maximum reduction is important. The limitation of this study is the small number of patients, 19 in each group, and it would be worth assessing these results on a larger group of people [69].

The search for biomarkers indicating biological and functional effects is ongoing to assess whether heating, rather than smoking, can reduce the development of chronic diseases. In one of such studies by Lüdicke et al., 8 biomarkers were assessed: HDL-C, white blood cell (WBC), forced expiratory volume in one second (FEV1) post-bronchodilator, expressed as % predicted (FEV1%pred), carboxyhemoglobin (COHb) in blood, total 4-(methylnitrosamino)-1-(3-pyridyl)-1-butanol (Total NNAL), soluble intercellular adhesion molecule-1 (sICAM-1) in serum, 11 dehydrothromboxane B2 (11-DTX-B2), 8-epi-prostaglandin F2 alpha (8-epi-PGF2α). After 6 months, there was a statistically significant improvement in 5 of the 8 biomarkers (HDL-C, WBC, FEV1% pred, COHb, total NNAL) in smokers who switched to HTPs compared to those who continued to smoke. However, it should be added that this study was conducted by the tobacco industry [70].

Akiyama and Sherwood published a comprehensive review of biological markers of tobacco-related exposure. The authors focused on the analysis of biomarkers of exposure (BOE) and biological effect (BOBE) for tobacco smoke. BOE levels measured in e-cigarette and HTP users were found to show a significant reduction compared to cigarette use. The authors suggested that a beneficial change in BOBE, including variables related to lipid metabolism, endothelial function, inflammation, oxidative stress, platelet activation, and lung function, could potentially contribute to improved health outcomes [71].

## Smoking cessation

Prevention plays a very important role in the approach to COPD. Quitting cigarette smoking appears to be the most important factor in reducing the incidence and mortality of COPD. Moreover, it is most important in restraining the progressive decline in lung function and the occurrence of exacerbations in people with COPD. Patients with a high degree of nicotine addiction to smoking have a huge problem with quitting smoking. It is estimated that up to 40% of patients diagnosed with COPD smoke continuously, even with severe disease [72]. Intensive smoking cessation programmes include strong educational support about the harmfulness of smoking and methods of dealing with the addiction, nicotine replacement therapy, and pharmacological treatment (bupropion, combined preparations with bupropion, varenicline, and cytisine) [73]. However, these programmes are poorly reimbursed and often overlooked by national healthcare systems. Tattan-Birch et al. performed a Cochrane Review on smoking cessation using HTPs. The authors included in the review 13 completed studies, of which 11 were RCTs assessing safety, and two were time series studies. The authors found no studies that reported the effectiveness of heated tobacco for smoking cessation [74].

## Gaps in knowledge and future directions

Available studies do not exhaust the subject of the role of HTP in the course of COPD, smoking cessation, reduction of morbidity and mortality. Braznell et al. conducted a systematic review that included 40 studies, 29 of which were related to the tobacco industry. In their review, the authors assessed that the conduct and reporting of interventional HTP clinical trials were poor in many respects and limited to examining the effects of short-term exposure. These trials are not sufficient to determine whether HTPs are beneficial to public health, meaning they may not provide a sound basis for tobacco control policy decisions. The main problems they highlight concern the inappropriate randomisation of the data, the lack of registration of trials, the publication of results in a timely manner and the selectivity of published results. According to the authors of the above analysis, there were no significant differences between the results from industry-associated and independent studies. However, the authors found that most industry-related studies were at high risk of biased results [75]. In the future, long-term studies with good methodology, on the effectiveness of smoking cessation methods using HTPs, are needed. Studies should be randomised, non-industry-funded trials to make a reliable assessment of HTPs.

Long-term studies with appropriate biomarkers and lung function tests would be needed to see if switching from combustible cigarettes to HTPs reduces damage and the number of exacerbations in people with COPD. It would be advisable to assess measures of lung function FEV1, FVC and FEV1/FVC, but also cardiovascular parameters such as blood pressure, heart rate, heart rate variability, and blood oxygen saturation [74]. Most studies have focused on comparing the toxic ingredients of different tobacco products. However, there is still little research on the immune effects of CSs use compared to non-combustible tobacco products. Most of these studies are not randomised, and the observation period is quite short. There is still a small number of clinical trials, and this area is worthy of attention. Due to the advances in molecular biology, research on the epigenetic mechanisms underlying the pathogenesis of COPD and the effects of tobacco smoke is emerging. However, there is still little information in the literature on this topic.

## Conclusion

Currently, available research results suggest that HTPs may play a role in harm reduction if smokers completely switch to HTPs from combustible cigarettes. It has been shown that there is less exposure to toxic substances like carbonyls or ROS using HTPs. These products also show better results in clinical trials with fewer exacerbations in COPD patients switching to HTPs compared to continuing smoking. The levels of tobacco exposure-related biomarkers were improved following HTP use, compared to smoking CSs. More long-term population-based studies are needed to look at the effects of HTPs, especially in COPD. Nevertheless, it should be emphasised that each tobacco product has a negative effect on lung tissue and can cause inflammation. There is no doubt that it is best to give up nicotine addiction entirely.

## Data Availability

Not applicable.
